# A case report of an epidermoid inclusion cyst following repair of hypospadias

**DOI:** 10.1016/j.ijscr.2024.109521

**Published:** 2024-03-20

**Authors:** Muddasir Fiaz Gondal, Rumaisaa Saman, Ali Raza Chaudhry, Hasnain Aslam, Salman Qamar, Waqas Sahi

**Affiliations:** a Paediatric Surgery Department, Holy Family Hospital, Rawalpindi, Pakistan; b Neurosurgery Department, Holy Family Hospital, Rawalpindi, Pakistan

**Keywords:** Epidermoid cyst, Urethrocutaneous fistula, Hypospadias repair, Penile cyst, Penis, Penile surgery

## Abstract

**Introduction and importance:**

Epidermoid cysts also known as epidermal inclusion cysts are the most common type of cutaneous cysts. These are derived from ectoderm with a lining of stratified squamous epithelium. Penile epidermoid cysts however are very rare. We report a case of Fifteen years old male with complain of slow growing mass at ventral aspect of shaft of penis along with urethrocutaneous fistula following hypospadias surgery. Surgical excision was done of the cyst. Histopathology of the sample revealed an epidermoid cyst.

**Case presentation:**

We report a case of a Fifteen years boy who underwent midshaft hypospadias repair at the age of five years. During postoperative period stent was removed and one week after that he developed a urethrocutaneous fisula. At seven years of age patient reported a small swelling on the penile shaft which gradually increased in size over the years, however, he seeks no medical care for it.

**Clinical discussion:**

At time of presentation swelling was separately appreciable from urehtrocuataneous fistula and extending from subcoroanal to midshaft of penis. We did excision of epidermal cyst and repair of urethrocutaneous fistula.

**Conclusions:**

Epidermal inclusion cyst as a complication of hypospadias surgery is a very rare situation. The diagnosis is made histologically and surgical excision is sufficient for treatment.

## Highlights

• Penile epidermoid cysts are rare after hypospadias repair and urethrocutaneous fistula is even more rare.
• Surgical management is the mainstay.
• The goal is to excise the cyst completely to avoid recurrence.
• Hypospadias surgery should be done in designated institutions by experienced surgeons to minimize such incidents and to achieve more favorable patient outcomes.
• Neoplastic transformation is rare but functioning may be affected because of the cyst location.

## 1 Introduction

Epidermal cysts are frequently observed over the body but rarely they are located in the penis. They are either congenital or acquired, with congenital being more common in pediatric age group. Congenital penile epidermal cysts form due to abnormal embryologic closure of the median raphe and therefore are also termed as “median raphe cysts” [1]. These cysts can occur anywhere along the line of penoscrotal raphe from urethral meatus till the anus. Acquired epidermal cysts occur as a result of trauma or surgical procedures due to implantation of epidermal epithelial cells in the dermis. As opposed to the median raphe cyst, epithelium of the epidermal cyst is lined with keratin. Özkan et al. reported the rate of inclusion cyst development after circumcision as 0.015 % in a series of 1900 cases. The surgical procedures often involved in formation of epidermal cyst as a complication are circumcision and hypospadias repair. A study showed post circumcision epidermoid cyst formation in 0.015 % of the cases among a total of 1900 cases [2]. Epidermoid cyst following hypospadias surgery is considered a rare occurrence. As per my literature review a total of around 30 cases has been reported so far, first case was reported in 2013 [3], followed by a case study including 24 cases published in 2022.

Here we report a case of epidermal cyst along with urethrocutaneous fistula that developed as a complication of hypospadias surgery. We declare that the work has been reported in line with the SCARE 2020 criteria [4].

## 2 Case presentation

**Fig. 1:**
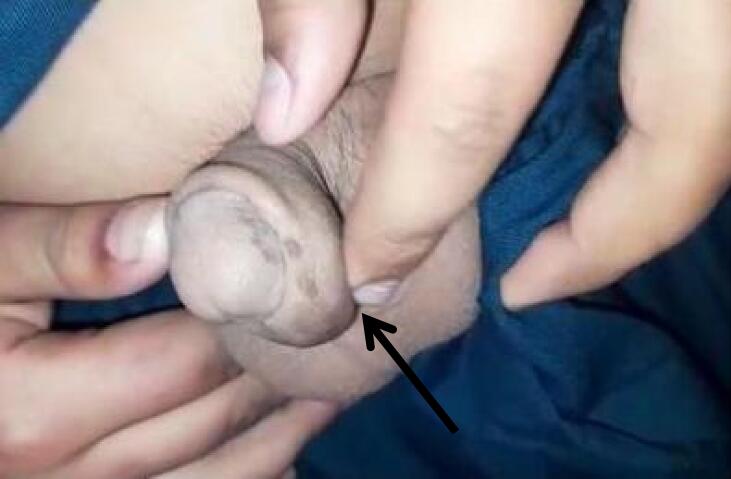
Pre-operative picture, black arrow pointing the cystic swelling on ventral aspect of penis.

**Fig. 2:**
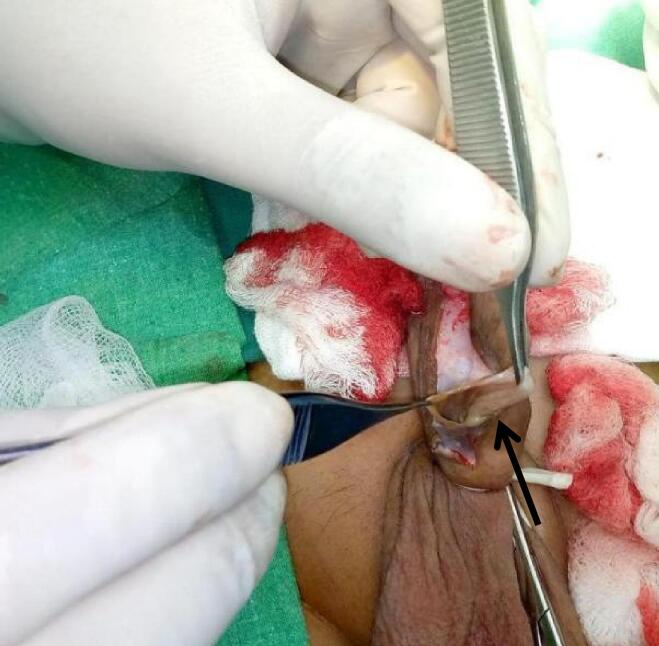
Black arrow pointing to the cyst.

**Fig. 3:**
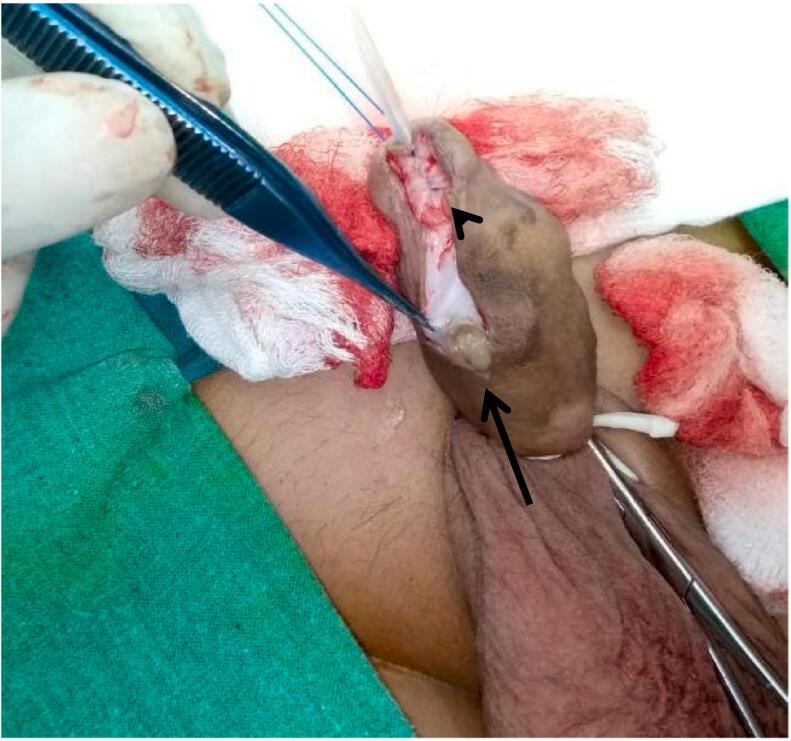
Black arrow head pointing the glandular meatal repair area, Black arrow pointing the area from where the cyst being removed.

We report a case of post hypospadias repair patient presented to us at fifteen years of age with a history of gradually increasing painless swelling on the penile shaft since the last eight years along with micturition from abnormal meatus. Previously he had surgery for distal shaft hypospadias at five years of age and post operatively he developed uretherocutaneous fistula. Physical examination revealed a fistula proximal to normal meatal opening with urine flow from the fistula and a 2 × 2 cm mass on ventral aspect of shaft of penis. As shown in Fig. 1. The mass was round, firm, nontender and freely mobile with no skin changes over the mass. Rest of the examination was unremarkable. Urine routine examination and culture were also unremarkable. Surgical management was planned and we performed Snodgrass repair along with excision of the cyst. Per operatively he is noted to have abnormal meatus at granular region along with a cystic swelling. Fig. 2 demonstrating cyst outline per operative while in Fig. 3 one can appreciate the snodgrass repaired area and part from where cyst is being excised. Foley's catheter placed and was removed on seventh post op day. Pt remains in follow-up till 3 months after the surgery and no complication or any symptom related to micturation was observed. Postoperative recovery was uneventful.

**Fig. 4:**
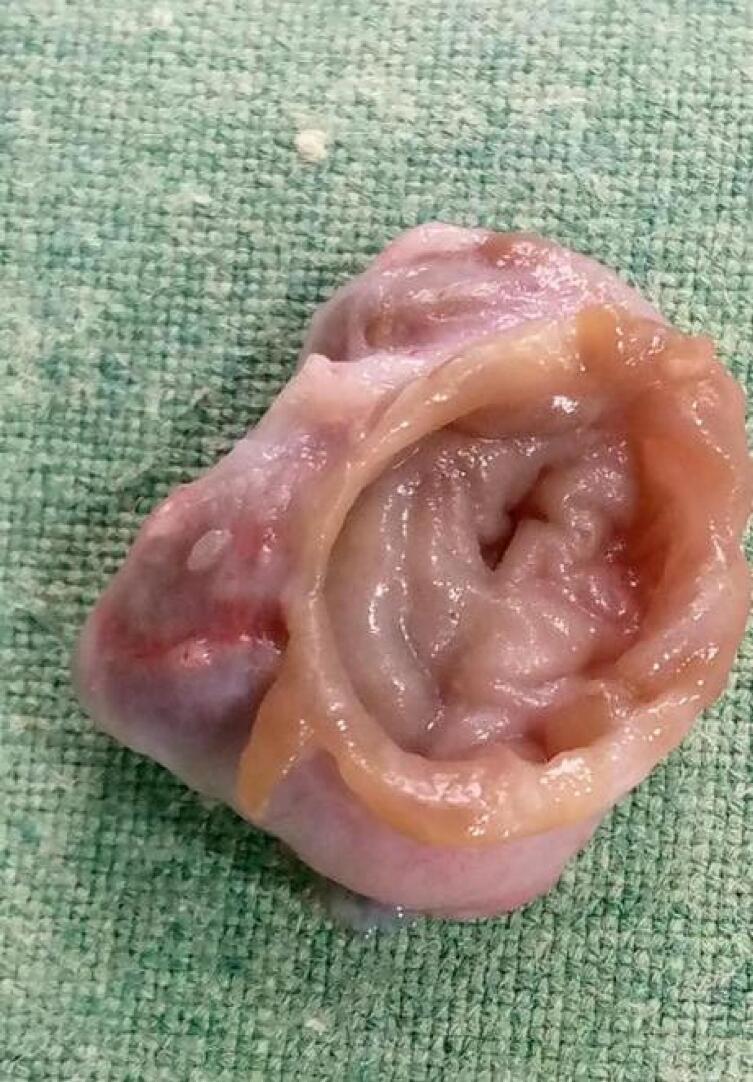
Excised cyst.

Fig. 4 shown the cyst excised in toto and the sample sent for histopathology. Histopathology of the sample revealed a benign cyst with extensive loss of epithelium with replacement by numerous foreign body type giant cells and numerous epithelioid cell collection, suggestive of epidermoid cyst. Fluid from the cyst was submitted for culture which yielded no organism on growth.

## 3 Discussion

Epidermoid cysts have also been termed as epidermal inclusion cysts, inclusion cysts, infundibular cysts and keratin cysts in literature [5]. These are common benign lesions arising from infundibular portions of the hair follicle as a result of plugging of the follicular orifice. They can occur on any part of the body commonly encountered on face, neck, head and trunk. Vulval cysts have also been reported especially in populations where female circumcision is practiced [6]. Epidermoid cysts are mainly asymptomatic but can become infected and present with signs and symptoms caused by the inflammation.

Penile epidermoid cyst are rare [7] and can be classified into congenital and acquired. Congenital epidermoid cysts occur during embryonic life due to an error in embryogenic development of the median raphe [8]. Penile median raphe cysts are therefore present along the median line in the ventral aspect of shaft of penis. Acquired cysts are a result of traumatic or surgical implantation of epithelial elements in the dermis or occlusion of pilosebaceous units [9]. Circumcision and hypospadias surgery are the frequently attributed procedures for epidermoid cysts formations post operatively [10]. These can occur anywhere in the penis. A study conducted by Yagmur et al. that collected data on post-surgical penile epidermoid cyst over fifteen years revealed 54 % of the penile cysts were ventral, 29 % were lateral, and 17 % were dorsal [11]. Epidermoid cysts of the penis can be single or multiple.

An epidermoid cyst can be diagnosed on physical examination but ultrasonography and MRI can be done as further investigations. Histological examination is necessary for confirmation of diagnosis. Treatment is timely complete surgical excision of the cyst with meticulous dissection. Care should be taken to avoid rupturing of the capsule to prevent recurrence and complications such as rupture of the cyst, infection or incomplete cyst removal. A case of epidermal inclusion cyst causing a urethrocutaneous fistula after urethroplasty has also been reported [12]. Inflammation caused by the epidermoid cyst is likely the reason for the subsequent urethrocutaneous fistula. In our case scenario there was coexistence of a urethrocutaenous fistula with epidermoid cyst with the latter being reported about two years after the formation of fistula. Neoplastic transformation is rare but has been reported in epidermoid cysts [13]. However, no case of malignancy has been reported in penile epidermoid cysts thus far. Follow-up after surgery should be done to look for any recurrence of cyst.

## 4 Conclusion

Penile epidermoid cysts are rare and should be considered as a differential in swelling over the penis. These occur post minor procedures such as circumcision or post hypospadias repair so care should be taken during these procedures to avoid seedling of epidermal components into the dermis or subcutaneous plane to avoid such complications.

## Consent

Written informed consent was obtained from the patient's parents/legal guardian for publication and any accompanying images. A copy of the written consent is available for review by the Editor-in-Chief of this journal on request.

## Ethical approval

Ethical approval was deemed unnecessary by our institutional ethical committee, as the paper is reporting a single case that emerged during normal practice.

## Funding

This research did not receive any specific grant from funding agencies in the public, commercial, or not-for-profit sectors.

## Author contribution

Muddasir Fiaz Gondal: Conceptualization, Writing-Reviewing and Editing.

Rumaisaa Saman: writing-Original draft preparation.

Ali Raza Chaudhry: Data curation.

Hasnain Aslam: Statistical expertise.

Salman Qamar: Writing-Reviewing.

Waqas Sahi: Proof Reading.

All authors read and approved the final manuscript.

## Guarantor

Muddasir Fiaz Gondal.

## Conflict of interest statement

The authors declare that they have no competing interests.

## References

[1] Shao I.-H., Chen T.-D., Shao H.-T., Chen H.-W. (2012). Male median raphe cysts: serial retrospective analysis and histopathological classification. Diagn. Pathol..

[2] Ozkan A., Ozorak A., Oruc M. (2012). Retrospective investigation complications in nineteen hundred cases of circumcision. Konuralp Med. J..

[3] Cimador M., Pensabene M., Sergio M., Catalano P., de Grazia E. (2013). Coverage of urethroplasty in pediatric hypospadias: randomized comparison between different flaps. Int. J. Urol. Off. J. Japan. Urol. Assoc..

[4] Sohrabi C., Mathew G., Maria N., Kerwan A., Franchi T., Agha R.A. (2023). The SCARE 2023 guideline: updating consensus Surgical CAse REport (SCARE) guidelines. Int. J. Surg. Lond. Engl..

[5] Weir C.B., St. Hilaire N.J. (2023).

[6] Birge O., Ozbey E.G., Arslan D., Erkan M.M., Demir F., Akgor U. (2015). Vulvar epidermoid cyst and type 2 radical genital mutilation. Case Rep. Obstet. Gynecol..

[7] Amaranathan A., Sinhasan S.P., Dasiah S.D. (2013). Median raphe cysts of the prepucial skin, with triple histological linings: a case report and review of the literature. J. Clin. Diagn. Res..

[8] Giambanco A., Pensabene M., Giuffrè M., Cimador M. (2013). Epidermal inclusion cyst of the penis after urethroplasty causing an urethro-cutaneous fistula: a first case report. Pediatr. Med. Chir..

[9] Ealai P.A., Yadav V.K., Vanjare H.A. (2015). Penile epidermal inclusion cyst: a rare location. Case Rep..

[10] Saini P., Mansoor M.N., Jalali S., Sharma A. (2010). Penile epidermal inclusion cyst. Indian J. Pediatr..

[11] Yagmur I., Tekin A., Bağcı U. (July 29, 2022). Acquired penile epidermoid cysts in children. Cureus.

[12] Giambanco A., Pensabene M., Giuffrè M., Cimador M. (2013). Epidermal inclusion cyst of the penis after urethroplasty causing an urethro-cutaneous fistula: a first case report. Pediatr. Med. Chir..

[13] Kim T., Kim J., Choi J., Jo T., Lee H.W., Jeong W. (2020). Squamous cell carcinoma arising from a long-standing epidermoid cyst of the back. Arch. Aesth. Plast. Surg..

